# Using all-cause mortality to define severe RV dilation with RV/LV volume ratio

**DOI:** 10.1038/s41598-018-25259-1

**Published:** 2018-05-08

**Authors:** Stephan P. L. Altmayer, Q. Joyce Han, Karima Addetia, Amit R. Patel, Paul R. Forfia, Yuchi Han

**Affiliations:** 1 Cardiovascular division, Department of Medicine, University of Pennsylvania, Philadelphia, PA USA; 2 CAPES Foundation, Ministry of Education of Brazil, Brasilia, DF Brazil; 3 Cardiovascular Division, Department of Medicine, University of Chicago, Chicago, IL USA; 4 Cardiovascular Division, Department of Medicine, Temple University, Philadelphia, PA USA

## Abstract

Right ventricular (RV) end-diastolic volume (EDV) to left ventricular (LV) EDV ratio using cardiovascular magnetic resonance imaging (CMR) is an important parameter for RV size evaluation in additional to indexed EDV. We explore the severity partition for RV dilation using mortality in a population of 62 patients with pulmonary hypertension (PH). Cine short-axis images were acquired with a 1.5 T MR scanner using a steady-state free precession sequence. The optimal cutoff to classify severe RV dilation was determined by a receiver-operating curve (ROC) analysis based on mortality. We further defined mild and moderate categories by the standard deviation distance between normal and severely dilated and found the categories RV dilation by RV/LV volume ratio to be “mild” (1.27–1.69), “moderate” (1.70–2.29) and “severe” (≥2.30). There were significant differences in RVEDV and RV ejection fraction between “mild”, “moderate” and “severe” groups (p < 0.001). The “severe” category had a significantly higher mortality when compared to the “non-severe” categories (p < 0.001) while there was no difference among the “non-severe” dilated groups. We have shown that severe RV dilation partition can be defined using mortality with RV/LV volume ratio, which offers an outcome based grading of the “severe” category of RV dilation.

## Introduction

Cardiovascular magnetic resonance (CMR) has been widely used to evaluate right ventricular (RV) volume and function^1^. The RV end-diastolic volume indexed to body surface area (BSA) (RVEDVi) is commonly used to quantitatively determine RV enlargement in patients with RV pressure and volume overload^2–4^. However, indexing to BSA is not perfect and RVEDVi does not account for the size of the adjacent left ventricle (LV) and may fail to detect dilation in patients with smaller hearts. In light of this limitation, we have shown that RV/LV volume ratio is a more sensitive parameter to detect RV dilation than RVEDVi^5^. In our previous work^5^, in a group 152 controls, we determined the normal range of the RV/LV volume ratio to be 0.906 to 1.266, and found that combining this parameter (RV/LV volume ratio ≥1.27) with RVEDVi yielded a higher detection of RV enlargement in a population with pulmonary arterial hypertension (PAH).

In the present study, we sought to propose the severity categories of RV dilation based on RV/LV volume ratio. Determining partition values is complicated and most approaches have significant limitations^6^. Many cutoff values used in cardiovascular imaging are arbitrarily derived from experience-based consensus of experts in the field or from percentile or standard deviation from normal values in a healthy population^6,7^. Ideally, severe abnormality should be determined based on the prediction of risks and prognosis of having that severe abnormality.

Our goal is to propose severe RV dilation cutoff values based on mortality risk in a cohort of patients with pulmonary hypertension (PH), a group of diseases that mainly affect the RV.

## Methods

### Subjects and follow-up

We retrospectively identified 62 PH patients from two institutions (University of Pennsylvania and University of Chicago) who had undergone CMR evaluation with at least 1-year clinical follow-up from the baseline CMR scan or died within 1 year after the CMR. All patients had documented elevated pulmonary arterial pressure and pulmonary vascular resistance and were grouped conforming to the WHO classification of pulmonary hypertension^8^. All participants gave written informed consent at the time of the CMR. The retrospective review of clinical data was approved by the IRB at University of Pennsylvania and University of Chicago with a waiver of consent. All methods were performed in accordance with our institutional guidelines and regulations.

Medication list was retrieved from the day of the CMR. Patient records were reviewed for 6-minute walk distance (6MWD) and N-terminal pro-hormone of brain natriuretic peptide (NT-proBNP) that were performed within 60 days of the CMR date. The 6-minute walk test (6MWT) was performed according to the recommended guidelines^9^.

### CMR

CMR images were acquired on a 1.5 T Siemens (Avanto, Siemens Health Systems, Erlangen, Germany) or a 1.5 T Philips scanner (Achieva, Best, Netherlands). Cine short-axis images were obtained using steady-state free precession sequence (voxel size of 1.9 × 1.9 × 8 mm^3^; TE = 1.05–1.5 ms; TR = 25–35 ms; flip angle = 55–75°; field of view = 280–320 × 280–400 mm^2^, temporal resolution = 25–35 ms; slice thickness = 8 mm; reconstruction phases = 25–30). Ventricular segmentation was performed using QMASS (Medis, Leiden, The Netherlands) by one observer following the Society for Cardiovascular Magnetic Resonance guidelines^10^. Data from intra- and inter-observer reproducibility were previously published^5^.

### Statistical analysis

Statistical analysis was performed using SPSS v.23 (IBM Corp, Armonk, NY). The cutoffs for RV enlargement for RVEDVi and RV/LV volume ratio were defined as values higher than two standard deviations (SD) from the mean in a control population: RVEDVi ≥104 ml/m^2^ for females, ≥113 ml/m^2^ for males, and RV/LV volume ratio ≥1.27 for both genders^5^. Shapiro-Wilk test was used to test for normality. CMR-derived parameters among groups were compared using Kruskal-Wallis test, except for RVEF (which is normally distributed). Differences between “severe” and “non-severe” categories were analyzed using two-sample, two-tailed Student’s t-test. Cox proportional hazards models were used to investigate the association of the severity of the RV/LV volume ratio and the risk of all-cause mortality. Multivariate analysis was performed with all variables with a p-value less than 0.10 in the univariate analysis. Survival analysis was performed using the Kaplan–Meier method and the log-rank test. A p-value < 0.05 was considered significant.

### Severity grading

Optimal cutoff value to define severe RV dilation using RV/LV volume ratio was identified using the receiver-operating curve (ROC) analysis. The point with the highest sensitivity and specificity to predict all-cause mortality in our population (minimal Euclidean distance) was defined as “severe” enlargement. The SD from the mean of the normal control to “severe enlargement” is then calculated. The upper limit of normal was defined as RV/LV volume ratio values ≥2 SD from our control population^5^. We further defined empirically the partition value for “moderate” to be halfway in SD distance from the mean of the “normal” and the “severe” groups. “Mild” group is then determined as > +2 SD of the normal and < moderate.

### Data availability

The datasets generated during and/or analyzed during the current study are available from the corresponding author on reasonable request.

## Results

### Patient characteristics

Fifty-seven patients were classified as group 1 (pulmonary arterial hypertension), four were group 4 (chronic thromboembolic pulmonary hypertension (CTEPH)) and one patient was group 5 (PH with multifactorial mechanisms). Most patients in our study were white females with PAH and were of New York Heart Association (NYHA) functional class II (Table 1). Most patients (96.8%) were on stable medical therapy for PH at the time of the CMR, only 2 patients (3.2%) were not taking any medications (one with a recent diagnosis of CTEPH prior to surgery and one with mild PAH). The majority of patients (n = 41, 66.1%) received PAH-specific combination therapy. Of those on monotherapy, 10 received a phosphodiesterase 5 inhibitor, 5 received a prostanoid, 3 received an endothelin receptor antagonist, and one patient received rioceguat.

**Table 1:** Demographic characteristics (n = 62).

Parameters	Mean ± SD or N(%)
Age	52.8 ± 14.7
Female gender	49 (79%)
**Ethnicity**	
White	41 (66.1%)
African-American	16 (25.8%)
Asian	3 (4.8%)
Hispanic	2 (3.2%)
**WHO classification of PH**	
Group 1 (PAH)	57 (92%)
Group 4 (thromboembolic)	4 (6%)
Group 5 (multifactorial)	1 (2%)
**NYHA functional class**	
I	14 (22.6%)
II	38 (61.3%)
III	10 (16.1%)
NT-proBNP (pg/mL)	896.6 ± 1063.4 (n = 60)
6-minute walk distance (m)	397.3 ± 119.6 (n = 56)
**Treatment**	
PAH-specific monotherapy	19 (30.6%)
PAH-specific combination therapy	41 (66.1%)
Calcium channel blocker	11 (17.7%)
Oxygen	8 (12.9%)
Anti-coagulant	12 (19.3%)

Data is presented as mean ± standard deviation or number (%).

PH = pulmonary hypertension; PAH = pulmonary arterial hypertension.

The majority of 6MWD and NT-proBNP were obtained on the same day of the CMR. Four patients were excluded from the analysis as the 6MWD and NT-proBNP were measured more than 60 days apart from the CMR date. Two patients could not perform 6MWT due to severe dyspnea or weakness. The mean time interval between 6MWT and CMR was 6 days.

There were 8 mortality events during follow-up: seven due to complications of right heart failure and one due to sepsis after an abdominal surgery. The mean RV/LV volume ratio for the entire PH population was 1.75 ± 0.69. There was no significant difference in RV/LV volume ratio between males and females (p = 0.54).

### Classification of dilation severity categories

**Figure 1:**
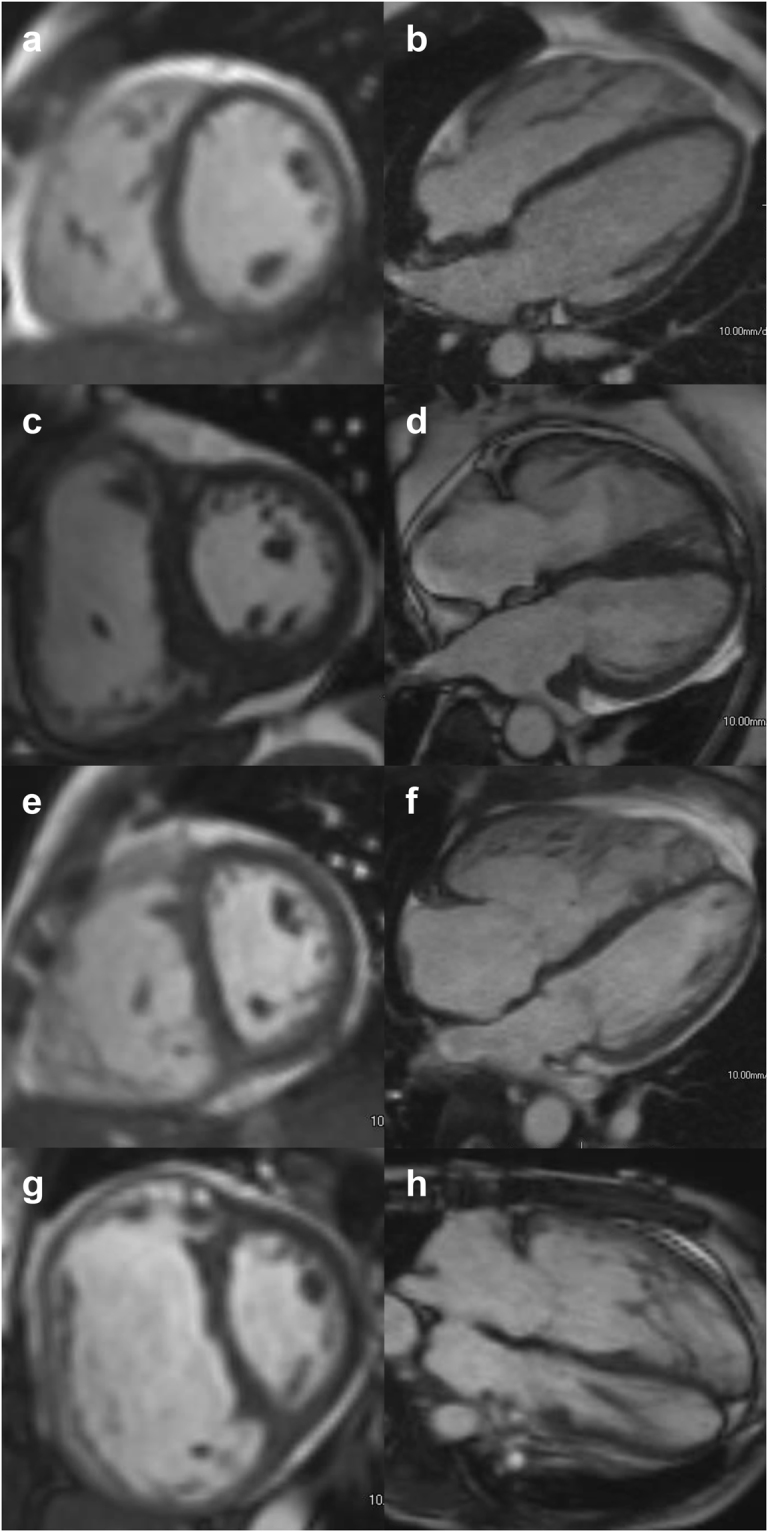
Examples of RV/LV volume ratio categories. (**a**,**b**) Short-axis (SAX) and 4-chamber views of a patient with a normal sized RV RV/LV volume ratio = 1.20); (**c**,**d**) SAX and 4-chamber views of a patient mildly dilated RV (RV/LV volume ratio = 1.50); (**e**,**f**) SAX and 4-chamber views of a patient with moderately dilated RV (RV/LV volume ratio = 1.70); (**g**,**h**) SAX and 4-chamber view of a patient with severely dilated RV (RV/LV volume ratio = 2.50).

**Figure 2:**
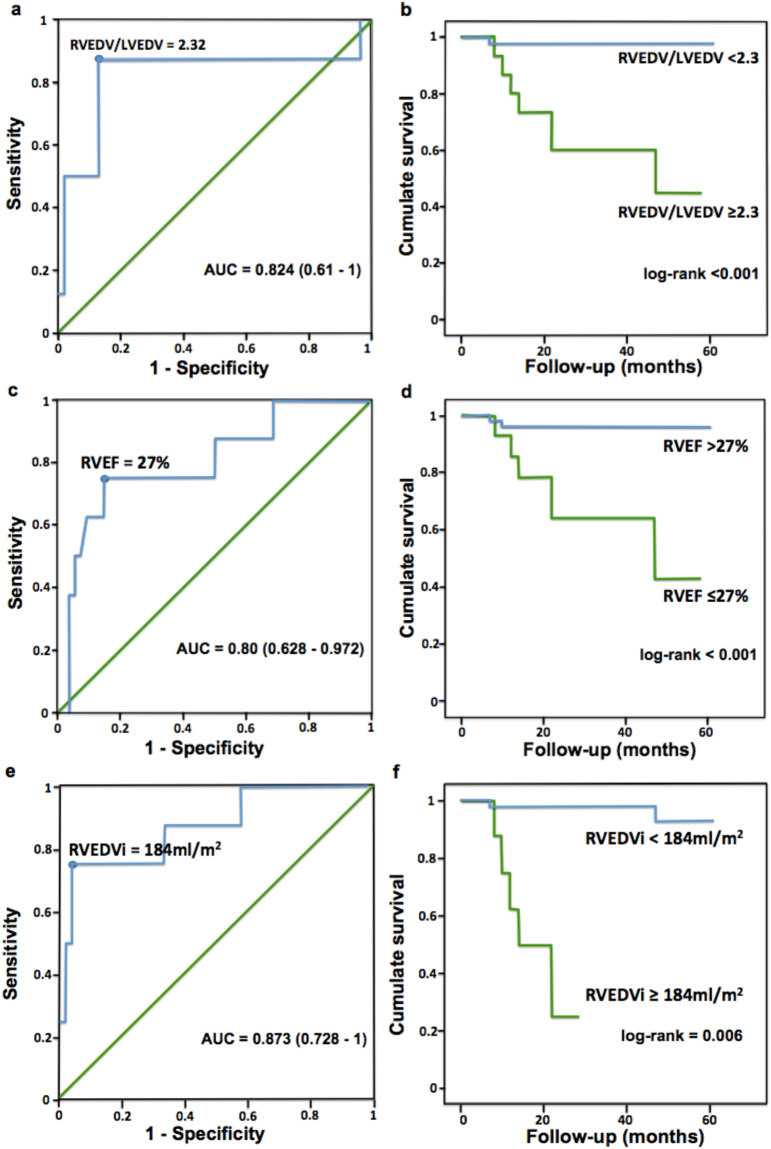
Receiver-operating curves and Kaplan-Meier survival analysis for RV/LV volume ratio, RVEDVi, and RVEF. (**a**) Optimal RV/LV volume ratio cutoff value to predict all-cause mortality. (**b**) The difference in event-free survival for “non-severe” vs. “severe” dilated RVs according to the RV/LV ratio. (**c**) Optimal RVEF cutoff value to predict all-cause mortality. (**d**) The difference in event-free survival for the optimal RVEF cutoff of 27%. (**e**) Optimal RVEDVi cutoff value to predict all-cause mortality. (**f**) The difference in event-free survival for “non-severe” vs. “severe” dilated RVs according to optimal cutoff for RVEDVi of 184 ml/m^2^.

Comparison of chamber size in end-diastole by RV/LV volume ratio in PH patients with different degrees of RV dilation is shown in Fig. 1. The ROC and Kaplan-Meier survival analysis for RV/LV volume ratio, RVEF and RVEDVi are shown in Fig. 2. The optimal cutoff value of RV/LV volume ratio to predict all-cause mortality was 2.32 (sensitivity = 87.5%; specificity = 87.0%) (Fig. 2a). Thus, we defined “severe” RV dilation as a ratio greater than 2.30, which corresponds to 13.4 SD from the mean in our control population. We further suggest using the 1.70 (6.7 SDs from the mean, half the SD distance from normal to severe in our control population) as the partition value to classify “moderate” as values between 1.70 and 2.29 and “mild” as between 1.27 and 1.69 (2 SD to 6.7 SD from the mean). This differentiation between mild and moderate remains arbitrary in our study and could be considered overall as “non-severe” dilation.

**Figure 3:**
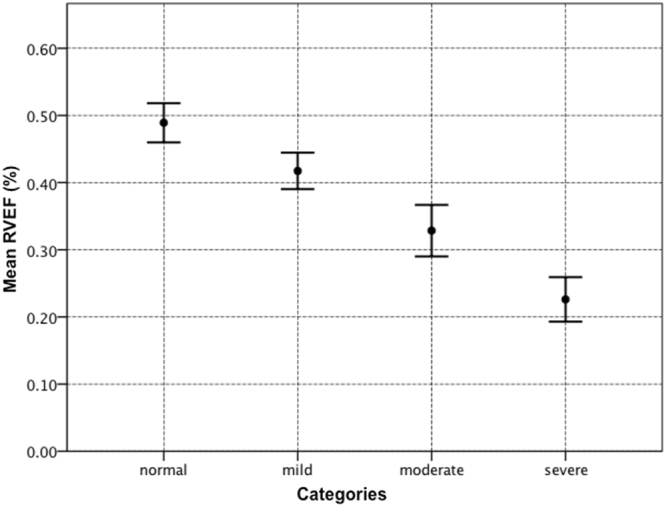
Mean RVEF stratified by RV/LV volume ratio severity. Error bars represent standard error of the mean for RVEF. The severity of RV dilation, represented by each category of the RV/LV volume ratio, was related to significantly higher degrees of RV dysfunction.

The CMR measurements for the RV according to our severity grading are shown as mean ± SD in Table 2. There were significant differences in RV/LV volume ratio, RVEDV, RVEDVi and RVEF between “mild”, “moderate” and “severe” groups (p < 0.001), but not for RV stroke volume (p = 0.27). The categories demonstrated significantly different degrees of RV dysfunction according to the severity of RV/LV volume ratio as shown in Fig. 3. The “severe” group had significantly higher values of NT-proBNP (1863.6 ± 1098 pg/mL, n = 15) compared to the “non-severe” patients (556.9 ± 819 pg/mL, n = 46; p = 0.001). Also, patients with “non-severe” dilation performed better in the 6MWT (417.5 ± 117.4 meters, n = 45) compared to the “severe” (354.3 ± 120.6 meters, n = 14; p = 0.086), although not statistically significant. In total, the RV/LV ratio alone classified 43 subjects (69.3%) of the study group as having any RV enlargement. Of those, it detected 10 patients with RV dilation that had a normal RVEDVi.

**Table 2:** CMR-derived RV/LV volume ratio, RV volumes, and EF stratified by category.

	Normal (n = 19)	Mild (n = 18)	Moderate (n = 10)	Severe (n = 15)
RVEDV/LVEDV	1.12 ± 0.11	1.43 ± 0.09	1.83 ± 0.18	2.67 ± 0.34
RVEDV (ml)	175.2 ± 57.0	201.1 ± 53.7	252.0 ± 42.5	340.1 ± 115.2
RVEDVi (ml/m^2^)	90.4 ± 18.6	112.8 ± 28.3	142.9 ± 27.5	191.3 ± 60.7
RVSV (ml)	84.2 ± 22.0	82.5 ± 19.7	82.2 ± 18.6	73.8 ± 21.6
RVEF (%)	48.9 ± 6.1	41.7 ± 5.8	32.8 ± 6.2	22.6 ± 6.5

Data is presented as mean ± standard deviation.

RVEDV, right ventricular end-diastolic volume; LVEDV, left ventricular end-diastolic volume; RVEDVi, right ventricular end-diastolic volume index; RVSV, right ventricular stroke volume; RVEF, right ventricular ejection fraction.

### Survival analysis

Survival analysis was performed comparing “severe” vs. “non-severe” groups for all variables. From the eight patients who died during the follow-up period, seven had an RV/LV volume ratio >2.3 and all died due to complications of RV failure. There was one death in a patient with normal RV size (RV/LV ratio <1.27), but it was due to a complication of an abdominal surgery. There was no statistical difference in mortality among the “non-severe” RV/LV volume ratio subgroups (p = 0.45). Event-free survival for the “severe” group was 80% at 1 year, 60% at 2 years, and 45% at 5 years (p < 0.01). Thus, the “severe” category had a statistically significant (p < 0.001) higher mortality when compared to the “non-severe” group (Fig. 2b). When compared to the patients with non-severe dilation, the “severely dilated” RV/LV volume ratio group was approximately 24 times more likely to die (HR = 24.1, 95% CI 2.97–196, p = 0.003). This association remained significant after adjusting for other variables in the multivariate analysis (Table 3). Also, there was a trend of increased mortality in patients with PAH related to connective tissue diseases.

**Table 3:** Cox regression analysis for prediction of all-cause mortality.

	Univariate analysis		Multivariate analysis	
	HR (95% CI)	*p*	HR (95% CI)	*p*
Age (years)	1.05 (0.99–1.12)	0.097	1.02 (0.93–1.12)	0.667
Male	1.35 (0.27–6.71)	0.712	—	—
Ethnicity	0.57 (0.15–2.11)	0.405	—	—
CTD-related PAH	2.82 (0.7–11.3)	0.144	—	—
Severe RVEDV/LVEDV	24.1 (2.97–196)	0.003	24.9 (1.18–527)	0.039
RVEDVi	1.02 (1.01–1.03)	0.000	1.03 (1.01–1.06)	0.008
RVEF	0.91 (0.84–0.98)	0.011	1.26 (1.07–1.49)	0.005
RVSV	1.00 (0.97–1.04)	0.601	—	—
LVEDVi	1.01 (0.98–1.06)	0.355	—	—
LVEF	0.86 (0.77–0.95)	0.004	0.83 (0.71–0.96)	0.016
LVSV	0.98 (0.94–1.02)	0.316	—	—
NT-proBNP	1 (1.00–1.01)	0.000	1 (1.00–1.002)	0.073
6MWT	0.99 (0.99–1.01)	0.105	—	—
**NYHA functional class**				
NYHA I	1	0.393	—	—
NYHA II	0.42 (0.08–2.1)	0.290	—	—
NYHA III	1.31 (0.21–8.0)	0.768	—	—

CTD, connective tissue disease; PAH, pulmonary artery hypertension; RVEDVi, right ventricular end-diastolic volume index; RVEF, right ventricular ejection fraction; RVSV, right ventricular stroke volume; LVEDVi, left ventricular end-diastolic volume index; LVEF, left ventricular ejection fraction; LVSV, left ventricular stroke volume; 6MWT, six-minute walk test; -, insignificant.

The optimal cutoff values for RVEF (Fig. 2c) and RVEDVi (Fig. 2e) in our patient population were 27% (sensitivity = 0.75; specificity = 0.85) and 184 ml/m^2^ (sensitivity = 0.75, specificity = 0.96), respectively. Event-free survival for the “severe” group according to RVEF (Fig. 2d) was 78% at 1 year, 50% at 2 years, and 36% at 5 years (p < 0.01). Survival for the patients in the “severe” group for RVEDVi (Fig. 2f) was 63% at 1 year and 25% at 2 years (p < 0.01).

## Discussion

We determined the “severe” enlargement of the RV using RV/LV volume ratio on the basis of the optimal cutoff values to predict all-cause mortality in a group of PH patients. The partition values proposed for “mild” (1.27–1.69), “moderate” (1.70–2.29), and “severe” (≥2.30) are able to successfully distinguish the categories in regard to the degree of RVEF impairment and all-cause mortality in our population. Also, patients in the “severe” category had significantly higher levels of NT-proBNP, which had been shown to be highly sensitive to predict mortality in patients with PH^11^.

Most of the partition values used in cardiovascular imaging, such as echocardiography-derived LV size, function, and LA volume, were empirically defined by consensus of experts’ opinions to provide a uniform reference that can be interpreted by clinicians^6^. However, the prognostic value of these semi-arbitrary categories is not well defined, as the cutoffs are not readily validated in separate cohorts^12^. For instance, recent guidelines for the diagnosis and treatment of PH established several cutoffs to aid the clinician to stratify the patient into “low”, “intermediate” or “high-risk”^13^. However, the guideline itself states that “most of the proposed variables and cut-offs are based on expert opinion”^13^; therefore, these categories may require further investigation to be validated. In our study, we used all-cause mortality in the PH cohort to define “severe” dilation.

All patients classified as “severe” by RVEDVi (n = 10, 16.1%) were also included in this category by the RV/LV volume ratio. However, the ratio classified 5 more patients into the “severe” group (n = 15) as compared to RVEDVi (n = 10), including an event of death. This shows the higher sensitivity of the “severely increased” RV/LV volume ratio when compared to the “severely increased” RVEDVi (87.5% vs. 75.0%) with a compromise of lower specificity (87.0% vs. 96.0%). Therefore, the RV/LV ratio is better at identifying patients with higher risk of all-cause mortality, but is less specific in predicting all-cause mortality than RVEDVi. Additionally, the same cutoff values of RV/LV volume ratio can be used for both males and females, as there is no significant gender difference in the RV/LV volume ratio^4,5^.

Recent studies on RV volume overload tried to propose cutoff values that integrate the severity of RV dilation and potentially irreversible RV remodeling. For instance, Lee *et al*. found that patients with tetralogy of Fallot (TOF) with a mean baseline RV/LV ratio of 2.2 demonstrated significant improvement in RV volume and function with pulmonary valve replacement^3^. Studies in the literature assessing RV dilation with pulmonic regurgitation had similar values (2.23^14^; 2.2^15^; 2.22^16^). Therefore, patients with “non-severe” RV dilation (defined as RV/LV volume ratio <2.2–2.3) are more likely to respond to medical therapy, which may explain the lower long-term mortality in this group.

From this perspective, appropriate interpretation of these categories is essential for clinical decision making in order to avoid further deterioration of RV function. Previous studies have already validated RVEF, RVEDVi, RV end-systolic volume index, and RV mass as predictors of mortality in PH patients in accordance with our findings^17–19^. Other CMR-derived functional parameters such as LVEDVi, LV mass, stroke volume index, and pulmonary artery relative area change have been described as prognostic markers in PH^18–21^. However, this is the first study to show that RV/LV volume ratio has a strong prognostic value in the evaluation of patients with PH. Moreover, this easily obtained parameter was also shown to predict significant complications related to RV dysfunction in other RV-disease populations such as TOF post-pulmonary valve replacement^4^.

Finally, we demonstrated that the RV/LV volume ratio improves the detection of RV dilation, which is consistent with our previous findings in a smaller cohort^5^. All 10 subjects considered as non-dilated RVs by the RVEDVi had “mild” dilation according to the ratio, which supports the idea that the RVEDVi may fail to detect dilation when the RV is enlarged compared to the adjacent LV. Thus, the patient’s LV is a more relevant point of indexing than BSA, because in addition to being representative as a point of reference for the individual, it also accounts for ventricular interdependence^22^. However, the RVEDVi remains essential for the RV evaluation in patients with biventricular dilation and those with “severely” enlarged RVs (RVEDVi ≥184 ml/m^2^), as it is a highly specific parameter to predict death since few patients in this category survived more than 24 months of follow-up, as shown in our study (Fig. 2f).

The retrospective design, small cohort size, and small number of events remain the limitations of our study. The cohort was mostly composed of patients with PAH and women, which reflects the female predominance in this disease^23^. Furthermore, this ratio will not be able to classify patients with biventricular dilation. In which case, RVEDVi alone will have to be relied upon. Larger prospective studies are needed to investigate the value of the RV/LV ratio categories in the long-term monitoring of patients and their responses to therapy.

## Conclusion

The RV/LV volume ratio of 2.3 or higher is associated with increased all-cause mortality in a PH population and should be considered as a cutoff for severe RV dilation. RV/LV ratio is an easily obtained, gender-independent, and a sensitive marker of RV dilation.
